# UBE4B Mediates Mitophagy via NIPSNAP1 Ubiquitination and NDP52 Recruitment

**DOI:** 10.3390/ijms27021119

**Published:** 2026-01-22

**Authors:** Bo Jin, Junyao Qu, Ke Xu, Yufei Zhang, Peng Xu, Xin Wang, Bo Zhao, Xianting Jiao

**Affiliations:** 1 Department of Pediatric infectious, Xinhua Hospital Affiliated to Shanghai Jiao Tong University School of Medicine, Shanghai 200092, China; 2 Engineering Research Center of Cell and Therapeutic Antibody, Ministry of Education, School of Pharmacy, Shanghai Jiao Tong University, Shanghai 200240, China

**Keywords:** ubiquitination, mitophagy, parkin, UBE4B, NIPSNAP1, HeLa cell

## Abstract

Mitophagy, as a critical form of selective autophagy, plays a central role in maintaining cellular homeostasis. While the canonical PTEN-Induced Kinase 1 (PINK1)–Parkin pathway is well established, mitophagy can still be effectively induced in Parkin-deficient cells such as HeLa, indicating the existence of Parkin-independent alternative pathways. The mitochondrial matrix proteins 4-Nitrophenylphosphatase domain and non-neuronal SNAP25-like protein homolog 1 (NIPSNAP1) acts as a key effector in such pathways, yet its regulatory mechanisms remain incompletely understood. Here, we identify Ubiquitination Factor E4B (UBE4B) as an E3 ubiquitin ligase for NIPSNAP1 and demonstrate that it catalyzes NIPSNAP1 ubiquitination in both Human Embryonic Kidney 293 cells (HEK293T) and HeLa cells. Under mitochondrial depolarization, UBE4B not only promotes NIPSNAP1 ubiquitination and subsequent lysosome-dependent degradation, but also significantly enhances its interaction with the autophagy adaptors Nuclear Dot Protein 52 kDa (NDP52) and Sequestosome 1 (p62/SQSTM1). Notably, while Parkin does not ubiquitinate NIPSNAP1, UBE4B-mediated ubiquitination facilitates mitophagy in Parkin-null HeLa cells by strengthening the binding between NIPSNAP1 and NDP52. Collectively, this study unveils a novel mitophagy pathway regulated by the UBE4B-NIPSNAP1 axis, offering new insights into mitochondrial quality control.

## 1. Introduction

Autophagy is a highly conserved catabolic process in which cellular components such as damaged organelles, misfolded proteins, or pathogens are enveloped by double-membrane vesicles called autophagosomes and delivered to lysosomes for degradation and recycling [1,2]. This process plays an essential role in maintaining cellular homeostasis, responding to nutrient stress, and clearing dysfunctional cellular components [3]. Dysregulation of autophagy has been closely linked to a number of major human diseases, including neurodegenerative disorders, cancer, infectious diseases, and aging [4]. In selective autophagy, receptor proteins such as Sequestosome 1 (p62, also known as SQSTM1) [5], Neighbor of BRCA1 gene 1 protein (NBR1) [6], Optineurin (OPTN) [7], Nuclear Dot Protein 52 kDa (NDP52) [8], and Tax1-Binding Protein 1 (TAX1BP1) [9] act as key mediators. These receptors simultaneously recognize ubiquitinated substrates and autophagic membranes, thereby targeting specific cargo to autophagosomes for degradation [10]. Among these, p62 is a canonical selective autophagy receptor. It binds to polyubiquitinated substrates—including damaged mitochondria, protein aggregates, and invading pathogens—through its C-terminal ubiquitin-associated (UBA) domain [11]. Concurrently, p62 interacts directly with Microtubule-Associated Protein 1A/1B-Light Chain 3 (LC3) proteins on the autophagosomal membrane via its LC3-interacting region (LIR) motif [5]. Furthermore, p62 can self-oligomerize through its N-terminal PB1 domain. This polymerization facilitates the clustering of ubiquitinated cargo into larger complexes, promoting their efficient sequestration and subsequent autophagic degradation [12]. Mitophagy, a critical form of selective autophagy, is responsible for the removal of dysfunctional or superfluous mitochondria. It plays an essential role in regulating mitochondrial quantity, sustaining energy metabolic homeostasis, and preventing excessive accumulation of reactive oxygen species (ROS) [13,14,15,16]. The canonical mitophagy pathway is orchestrated by a signaling cascade mediated by the serine/threonine kinase PINK1 and the E3 ubiquitin ligase Parkin. Following mitochondrial depolarization, PINK1 accumulates on the outer mitochondrial membrane (OMM), where it phosphorylates ubiquitin and recruits/activates Parkin, triggering the latter’s E3 ubiquitin ligase activity [17,18]. Activated Parkin subsequently ubiquitinates multiple OMM proteins, generating ubiquitin chains that serve as recognition signals for autophagy receptors such as p62, OPTN, and NDP52 [7,19,20,21]. This cascade culminates in the engulfment and degradation of damaged mitochondria by autophagosomes. Mutations in the PINK1-Parkin pathway are a major cause of familial Parkinson’s disease, underscoring its critical importance in the pathogenesis of neurodegenerative disorders [22,23]. However, it is noteworthy that robust mitophagy can still be induced in cell types lacking Parkin expression, such as HeLa cells. This evidence indicates the existence of alternative, Parkin-independent mitophagy pathways, the molecular mechanisms of which remain to be fully elucidated [24,25].

The Nitrophorin-associated Insect Protein Secreted from Neuronal Axons and Peripheral Tissues (NIPSNAP) protein family comprises evolutionarily conserved proteins widely present in eukaryotes and represents one of the key regulators of alternative mitophagy pathways [26,27]. In mammals, this family primarily consists of two paralogous members, NIPSNAP1 and NIPSNAP2, which arose from gene duplication events and share high sequence and structural similarity. Both play essential roles in mitochondrial function and quality control [28,29]. Under physiological conditions, NIPSNAP proteins are primarily localized to the mitochondrial matrix or inner membrane. Upon mitochondrial damage and depolarization, they translocate and accumulate on the outer mitochondrial membrane, where their surface exposure serves as an autophagy signal. These exposed NIPSNAP proteins directly recruit cytosolic autophagy receptors such as p62 and NDP52, thereby initiating a Parkin-independent mitophagy pathway [29]. This mechanism provides cells with an important alternative pathway for the clearance of damaged mitochondria [21].

Although the role of NIPSNAP1 in recruiting the mitophagy machinery during Parkin-independent pathways has been largely established, the precise regulation of its own activity remains unclear. Protein ubiquitination, a key post-translational modification, involves the sequential action of E1 ubiquitin-activating, E2 ubiquitin-conjugating, and E3 ubiquitin-ligating enzymes to covalently attach ubiquitin—a small 76-amino acid protein tag—to substrate proteins [30]. Ubiquitination not only directs substrates for degradation via the proteasome or lysosomal pathways but also serves as a versatile signaling mechanism that regulates a wide array of cellular processes [31]. Nevertheless, owing to the vast diversity of E3 ligases and their complex substrate recognition mechanisms, the identification of specific physiological substrates for individual E3s remains a major challenge in the field [32,33]. To address this challenge, our group previously developed the Orthogonal Ubiquitin Transfer (OUT) pathway for the systematic identification of direct substrates of specific E3 ubiquitin ligases. This engineered system operates independently of the endogenous ubiquitination cascade. In this approach, the E3 ligase is mutated to ablate its catalytic activity while preserving its substrate-binding capability, thereby enabling efficient and specific capture of its direct substrates [34]. Utilizing the Orthogonal Ubiquitin Transfer (OUT) platform, we have successfully identified potential substrates of multiple E3 ligases, including C-terminus of Hsc70-Interacting Protein (CHIP) [35,36], Ubiquitination Factor E4B (UBE4B) [35,37], Reverses Spt- Phenotype 5 (Rsp5) [38], and E6-Associated Protein (E6AP) [39,40,41]. UBE4B, also referred to as Ubiquitin Fusion Degradation 2 Homolog A (UFD2A), is a highly conserved eukaryotic protein [42]. UBE4B has been implicated in regulating diverse cellular processes, including the cell cycle, DNA damage response, and protein homeostasis, and its dysfunction is associated with various pathologies such as neurodegenerative diseases and cancer [43,44,45,46]. Notably, several key proteins, including p53, Ataxin-3, and FAT4, have been identified as canonical substrates of UBE4B [43]. In our previous screening of HEK293T cells using the OUT pathway, NIPSNAP1 was identified as one of over 100 potential substrates of UBE4B [35]. Building on this finding, the present study aims to validate the interaction between UBE4B and NIPSNAP1 and to investigate the functional role of UBE4B-mediated ubiquitination of NIPSNAP1 in mitophagy. These results indicate that ubiquitination of NIPSNAP1 by UBE4B enhances the recruitment of autophagy receptors, thereby facilitating the efficient initiation of mitophagy. This work reveals a Parkin-independent regulatory mechanism, providing new insight into the complex molecular orchestration of mitochondrial quality control.

## 2. Results

### 2.1. UBE4B Specifically Interacts with NIPSNAP1

**Figure 1:**
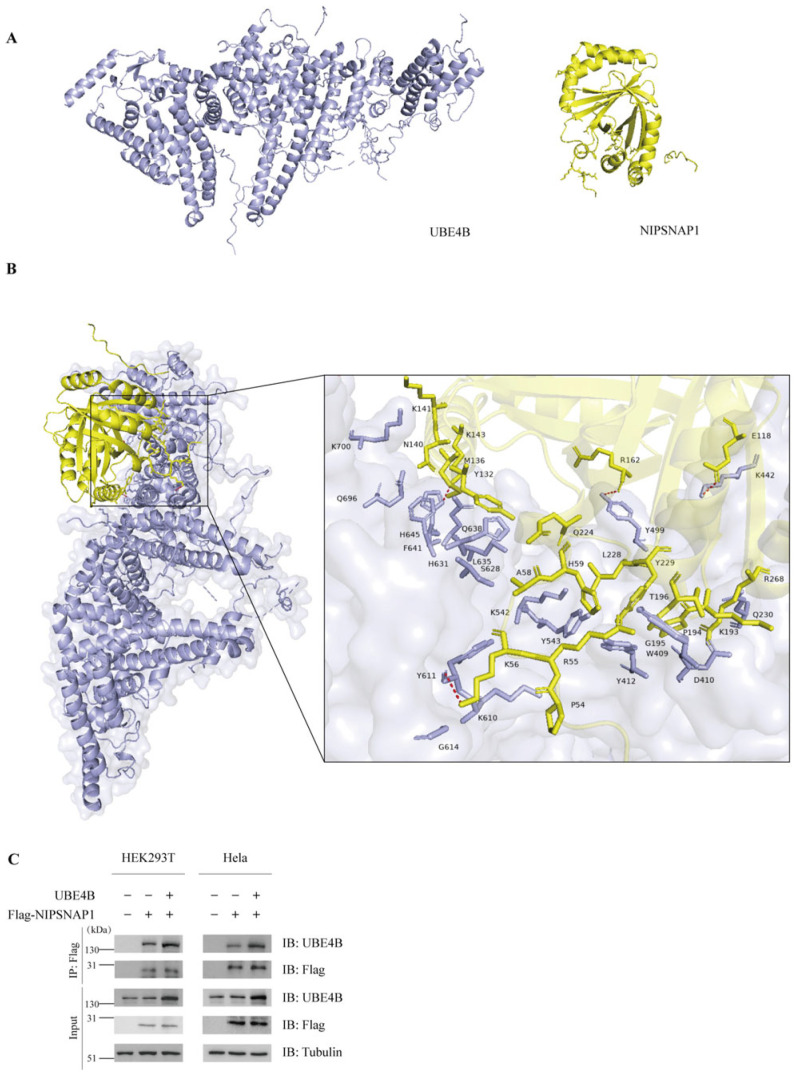
UBE4B interacts with NIPSNAP1. (**A**), Predicted three-dimensional structures of UBE4B (left, colored in purple) and NIPSNAP1 (right, colored in yellow) generated by AlphaFold3. (**B**), The structure of UBE4B sequence and overall view of the highest-ranked docking model of the UBE4B-NIPSNAP1 complex. UBE4B is shown in purple and NIPSNAP1 is shown in yellow. The predicted interaction interface is indicated by a dashed lines. (**C**), HEK293T and HeLa cells are cotransfected with FLAG-NIPSNAP1 (2 μg) and exogenous UBE4B (2 μg) or an empty vector (2 μg) plasmid as a control for 48 h. Cells without FLAG-NIPSNAP1 are used as a negative control. The interaction between UBE4B and NIPSNAP1 is detected by a coimmunoprecipitation assay with an anti-FLAG antibody and an anti-UBE4B antibody, respectively. The tubulin levels are used as an internal reference.

In a prior study, we identified NIPSNAP1 as a potential ubiquitination substrate of UBE4B. To verify this, we first investigated whether UBE4B and NIPSNAP1 physically interact. We began with computational docking analysis. The UBE4B protein primarily contains a U-box domain and a CUE domain. The U-box serves as the catalytic core, structurally and functionally resembling the RING-finger domain, and is responsible for recruiting E2-ubiquitin complexes [47,48]. The CUE domain functions as a ubiquitin-binding module that specifically recognizes and binds ubiquitin molecules already conjugated to substrate proteins, which is essential for UBE4B’s E4 activity [49]. Since the three-dimensional structures of these two proteins have not been publicly reported, we generated their structural models using AlphaFold3 (https://alphafoldserver.com/, accessed on 25 November 2025) (Figure 1A). Given that the structure of Ufd2, the yeast homolog of UBE4B, has previously been determined [50], we aligned the sequences of both proteins to infer the approximate domain boundaries within UBE4B. The resulting predicted structure exhibited significant similarity to the yeast template, suggesting a high degree of reliability for our model. Subsequently, we employed the H-DOCK server to perform molecular docking calculations using these predicted structures [38,51,52,53,54,55]. The analysis yielded favorable docking metrics (Table 1), where lower (more negative) docking scores generally indicate stronger predicted binding affinity and higher complex stability. Notably, the top two predicted models displayed docking scores below −240 and confidence scores exceeding 85%, indicative of a highly stable interaction. Although a lower RMSD value typically signifies better conformational stability—with the Rank 9 model showing the lowest RMSD (42.37 Å), suggesting a ligand position closer to the reference—the Rank 1 model demonstrated the lowest docking score combined with a high confidence score. Consequently, despite its higher RMSD, the Rank 1 model was identified as the most plausible configuration (Figure 1B). Specifically, the docking results revealed that NIPSNAP1 interacts with the UFD domain of UBE4B, which functions as the substrate-binding domain. These supportive scores, together with the complementary shapes and physicochemical properties observed at the predicted binding interface, provide computational evidence that the interaction is robust. In the visualized model, hydrogen bond interactions are highlighted by red dashed lines. Subsequently, we conducted co-immunoprecipitation (Co-IP) assays to examine the interaction between UBE4B and NIPSNAP1 in cells. HEK293T and Hela cells were cotransfected with FLAG-tagged NIPSNAP1 and exogenous UBE4B. Control cells were transfected with FLAG-NIPSNAP1 and an empty vector, or with the empty vector alone. After pulldown with an anti-FLAG antibody, an anti-UBE4B antibody was used to detect the interaction. Due to the presence of endogenous UBE4B, weak bands were observed in control cells transfected only with FLAG-NIPSNAP1, while no bands were detected in cells transfected with the empty vector alone. In contrast, cells cotransfected with exogenous UBE4B and FLAG-NIPSNAP1 showed significantly stronger bands, indicating a specific interaction between UBE4B and NIPSNAP1 (Figure 1C). UBE4B was reliably detected in the immunoprecipitates, confirming that exogenously expressed UBE4B interacts with NIPSNAP1 within cells.

### 2.2. UBE4B Mediates the Ubiquitination of NIPSNAP1

**Figure 2:**
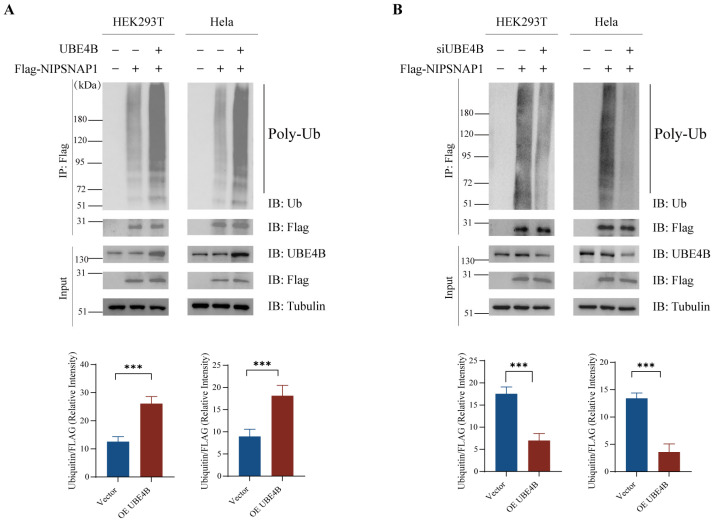
UBE4B promotes NIPSNAP1 ubiquitination. (**A**), HEK293T and HeLa cells are cotransfected with FLAG-NIPSANP1 (2 μg) and exogenous UBE4B (2 μg) or an empty vector (2 μg) plasmid as a control for 48h and then treated with MG132 (10 μM) for 4h. NIPSNAP1 ubiquitination is detected by a coimmunoprecipitation assay with the anti-FLAG and anti-ubiquitin antibodies. (**B**), HEK293T and HeLa cells are cotransfected with siUBE4B or siNC as a control for 48 h and then treated with MG132 (10 μM) for 4 h. NIPSNAP1 ubiquitination is detected by a coimmunoprecipitation assay with the anti-FLAG and anti-ubiquitin antibodies. Data represent mean ± SD from three independent experiments. Protein levels were quantified using ImageJ (v1.54g) and normalized to tubulin. Statistical markers: *** *p* < 0.001.

To investigate whether UBE4B regulates the ubiquitination of its interacting protein NIPSNAP1, we performed the co-immunoprecipitation assay. Poly-ubiquitin bands were observed on NIPSNAP1, and these bands were markedly enhanced in cells overexpressing UBE4B, suggesting that the ubiquitination of NIPSNAP1 is mediated by UBE4B (Figure 2A). Conversely, knockdown of endogenous UBE4B by small interfering RNA markedly reduced ubiquitin conjugation on NIPSNAP1. These mutually supportive results, consistently observed in two independent cell lines, demonstrate that UBE4B promotes NIPSNAP1 ubiquitination.

### 2.3. Under Conditions of Mitochondrial Depolarization, UBE4B Enhances Endogenous NIPSNAP1 Ubiquitination and Promotes Its Degradation

**Figure 3:**
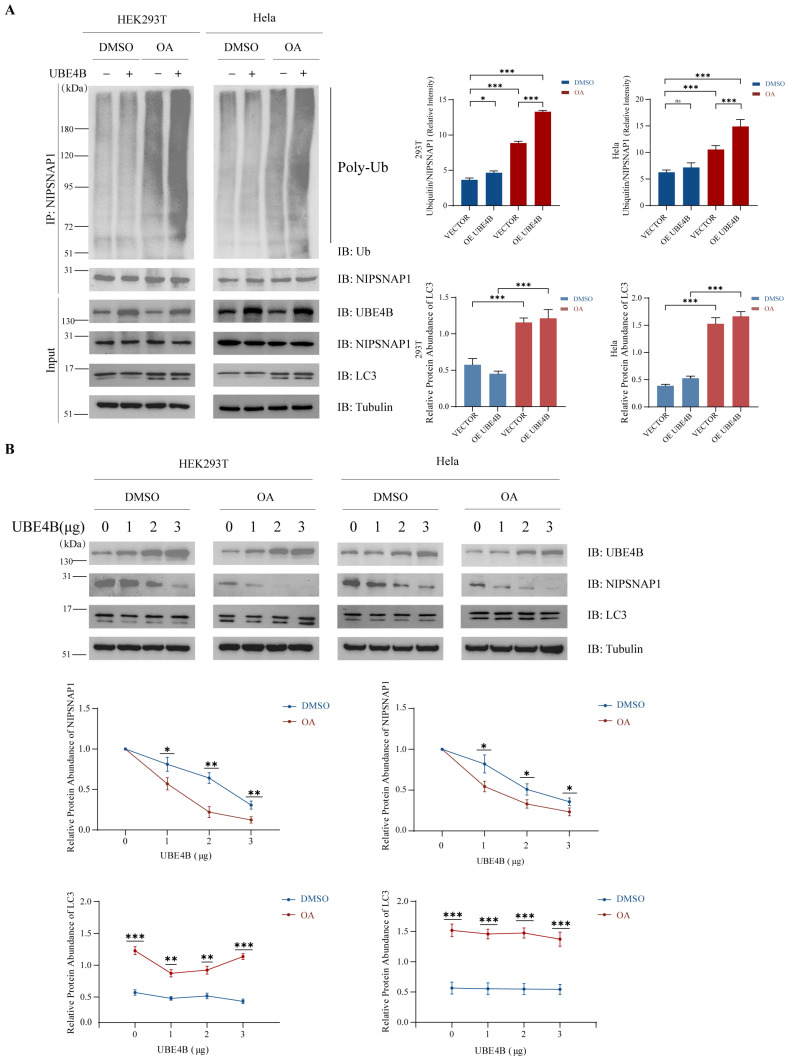
UBE4B mediates endogenous NIPSNAP1 ubiquitination and degradation upon mitochondrial depolarization. (**A**), HEK293T and HeLa cells are cotransfected with exogenous UBE4B (2 μg) or an empty vector (2 μg) plasmid as a control for 48 h and then treated with OA (10 μM Oligomycin plus 4 μM Antimycin-A) for 24 h, together with MG132 (10 μM) for 4 h. NIPSNAP1 ubiquitination is detected by a coimmunoprecipitation assay with the anti-NIPSNAP1 and anti-ubiquitin antibodies. (**B**), HEK293T and HeLa cells are transfected with different amounts of exogenous UBE4B for 48 h, and treated with OA (10 μM Oligomycin plus 4 μM Antimycin-A) for 24 h. NIPSNAP1 degradation caused by UBE4B is detected by Western blotting with the anti-UBE4B and anti-NIPSNAP1 antibodies. Data represent mean ± SD from three independent experiments. Protein levels were quantified using ImageJ and normalized to tubulin. Statistical markers: * *p* < 0.05; ** *p* < 0.01; *** *p* < 0.001; ns = not significant.

Given that NIPSNAP1 translocates to the mitochondrial outer membrane—the site of its ubiquitination—only upon mitophagy induction, we therefore performed subsequent experiments under depolarization conditions to investigated whether UBE4B-mediated regulation of NIPSNAP1 is activated. HEK293T and HeLa cells were treated with the mitophagy inducer a combination of Oligomycin and Antimycin A (OA) with control groups receiving equivalent DMSO treatment. We performed the co-immunoprecipitation assay to examine UBE4B’s effect on endogenous NIPSNAP1. Basal levels of mitophagy are present in cells; therefore, under unstimulated conditions (in the absence of OA stimulation), low levels of NIPSNAP1 protein are present on the outer mitochondrial membrane, and these proteins undergo ubiquitination. Under these conditions, overexpression of UBE4B resulted in a modest increase in polyubiquitination levels in HEK293T cells, whereas no significant change was observed in HeLa cells. Following OA treatment, NIPSNAP1 translocated from the mitochondrial interior to the outer membrane. Notably, under OA-induced conditions, UBE4B overexpression markedly enhanced ubiquitination signals in both cells (Figure 3A). To assess whether UBE4B-mediated ubiquitination triggers NIPSNAP1 proteolysis, we transfected cells with escalating amounts of UBE4B and monitored endogenous NIPSNAP1 levels. NIPSNAP1 protein abundance declined dose-dependently upon UBE4B overexpression, with greater UBE4B expression correlating with reduced NIPSNAP1 retention. Notably, this degradation was substantially accelerated under oligomycin/antimycin A (OA) treatment (Figure 3B), confirming that UBE4B-driven ubiquitination and subsequent degradation of NIPSNAP1 are activated during mitochondrial depolarization.

Concurrently, we assessed the lipidation status of LC3, a pivotal marker of autophagy. The accumulation of lipidated LC3 (LC3-II) serves as a canonical indicator of autophagosome formation [56]. As depicted in Figure 3A, compared with empty vector controls, UBE4B overexpression combined with OA treatment induced significantly enhanced LC3 lipidation in both cell lines. This robust autophagic activation, synchronized with NIPSNAP1 degradation, provides compelling evidence that UBE4B overexpression potentiates the progression of mitophagy.

### 2.4. UBE4B-Mediated Degradation of NIPSNAP1 Occurs via the Lysosome-Dependent Pathway

**Figure 4:**
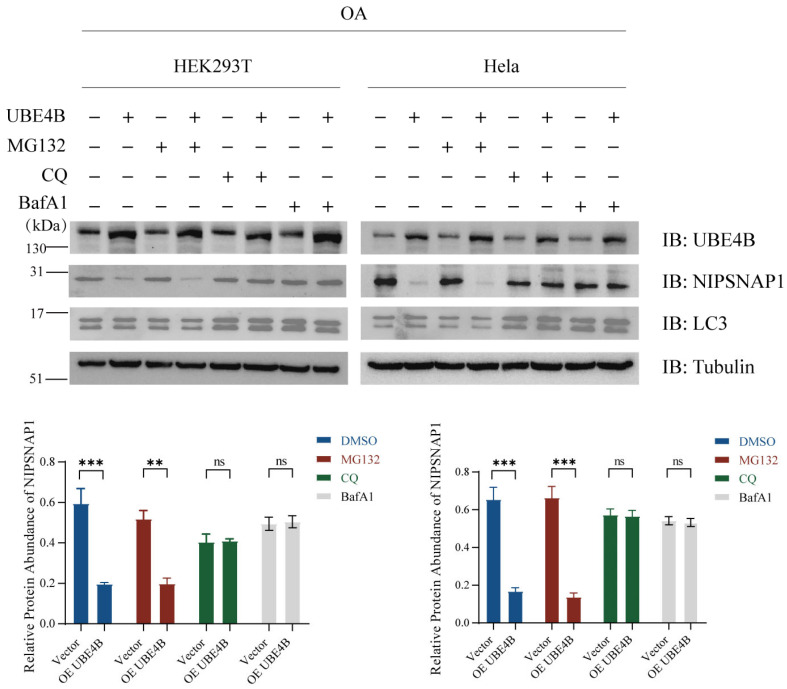
UBE4B-mediated degradation of NIPSNAP1 occurs via the lysosome-dependent pathway. HEK293T and HeLa cells are transfected with exogenous UBE4B (2 μg) or an empty vector (2 μg) plasmid and treated with OA (10 μM Oligomycin plus 4 μM Antimycin-A) for 24 h, together with MG132 (10 μM, 4 h), or chloroquine (CQ, 10 μM, 4 h), or BafA1 (10 μM, 4 h), and NIPSNAP1 degradation caused by UBE4B is detected by Western blotting with the anti-UBE4B and anti-NIPSNAP1 antibodies. LC3 is detected by Western blotting with the anti-LC3 antibody. Data represent mean ± SD (*n* = 3 independent experiments). Statistical analysis was performed using two-tailed Student’s *t*-test, all analyses performed using GraphPad Prism 8. Statistical markers: ** *p* < 0.01; *** *p* < 0.001; ns = not significant.

While prior experiments demonstrated that UBE4B promotes NIPSNAP1 degradation under mitochondrial depolarization, the specific degradation route remained unclear. To determine whether this occurs via lysosome-mediated autophagy or the proteasome pathway, we employed pharmacological inhibitors targeting these distinct degradation systems. We treated OA-stimulated HEK293T and HeLa cells with the proteasome inhibitor MG132, the lysosome inhibitor chloroquine (CQ), or the autophagy inhibitor bafilomycin A1 (BafA1). As shown in Figure 4, under conditions of UBE4B overexpression and induced mitophagy, detection of endogenous protein levels revealed that both CQ and BafA1 effectively blocked the UBE4B-driven degradation of NIPSNAP1, leading to its significant accumulation. These results clearly demonstrate that UBE4B predominantly promotes the degradation of NIPSNAP1 through the lysosome-dependent pathway, and we speculate this process involved in mitophagy.

### 2.5. Parkin Does Not Mediate the Ubiquitination of NIPSNAP1

**Figure 5:**
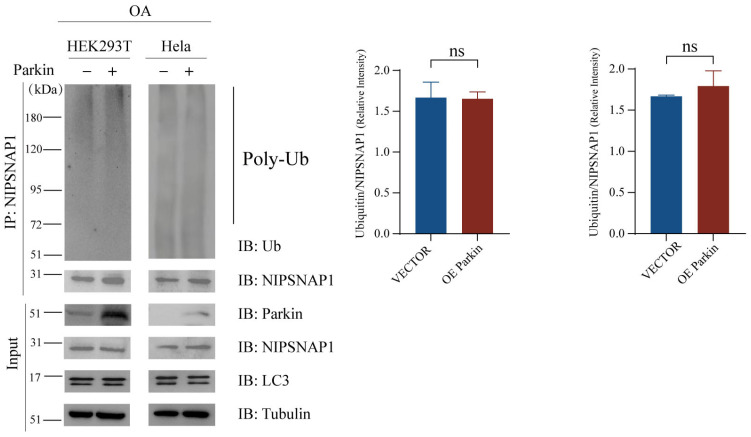
Parkin cannot mediate the ubiquitination of NIPSNAP1. HEK293T and HeLa cells are cotransfected with exogenous Parkin (2 μg) or an empty vector (2 μg) plasmid as a control for 48 h and then treated with OA (10 μM Oligomycin plus 4 μM Antimycin-A) for 24 h, together with MG132 (10 μM) for 4 h. NIPSNAP1 ubiquitination is detected by a coimmunoprecipitation assay with the anti-NIPSNAP1 and anti-ubiquitin antibodies. Data represent mean ± SD (*n* = 3 independent experiments). Statistical analysis was performed using two-tailed Student’s *t*-test, all analyses performed using GraphPad Prism 8. Statistical markers: ns = not significant.

Our previous findings demonstrated that UBE4B overexpression effectively promotes NIPSNAP1 lysosomal degradation and induces lipidated LC3 accumulation even in Parkin-deficient HeLa cells. This key evidence indicates that the UBE4B-driven degradation pathway operates independently of Parkin in this cell model. However, in HEK293T cells expressing endogenous Parkin, potential cooperation between UBE4B and the Parkin pathway could not be excluded. To directly examine whether Parkin ubiquitinates NIPSNAP1, we performed co-immunoprecipitation assays in both HEK293T and HeLa cells under OA treatment. Cells were transfected with Parkin plasmid, and lysosome inhibitors were applied to facilitate the accumulation of ubiquitinated proteins. Subsequent immunoprecipitation with an anti-NIPSNAP1 antibody revealed no enhancement of ubiquitin bands on NIPSNAP1 in Parkin-overexpressing cells compared to controls (Figure 5). These results demonstrate that Parkin does not mediate the ubiquitination of NIPSNAP1, further confirming that UBE4B promotes the degradation through a Parkin-independent mechanism by directly ubiquitinating NIPSNAP1. The ubiquitination of Rab5a by Parkin served as a positive control (Figure S1) [57].

### 2.6. UBE4B Enhances the Interaction Between NIPSNAP1 and Autophagy Receptors

**Figure 6:**
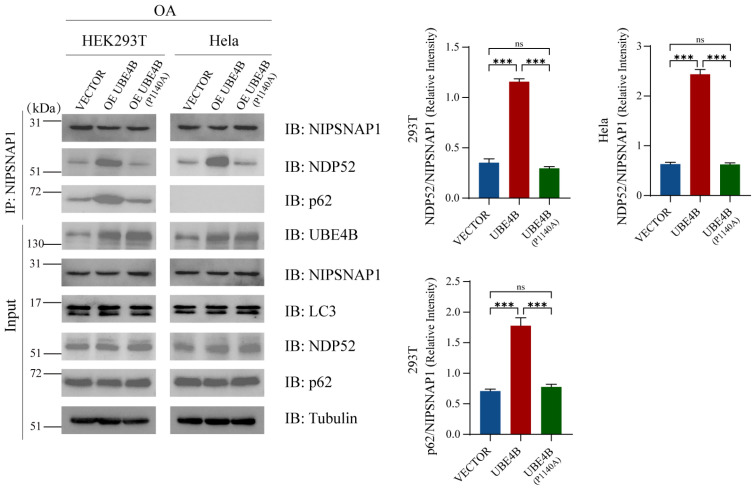
NIPSNAP1 interacts with NDP52 and p62. HEK293T and HeLa cells are cotransfected with one of following plasmids (2 μg each) for 48 h: exogenous UBE4B, the UBE4B mutant (P1140A) or an empty vector (as a control), followed by treatment with OA (10 μM Oligomycin plus 4 μM Antimycin-A) for 24h. The interaction between NIPSNAP1 and NDP52 or p62 are detected by a coimmunoprecipitation assay. Data represent mean ± SD (*n* = 3 independent experiments). Statistical analysis was performed using two-tailed Student’s *t*-test, all analyses performed using GraphPad Prism 8. Statistical markers: *** *p* < 0.001; ns = not significant.

To elucidate the regulatory role of UBE4B-mediated NIPSNAP1 ubiquitination in mitophagy, we further investigated the impact of UBE4B on the interaction between NIPSNAP1 and selective autophagy receptors. Previous studies have established that in the absence of Parkin, p62 is not effectively recruited to damaged mitochondria, whereas the recruitment of OPTN and NDP52 remains intact [21,58]. Additionally, in Parkin-proficient cell lines, NIPSNAP1 has been reported to recruit both p62 and NDP52 [29]. Based on these precedents, p62 and NDP52 were selected as representative autophagy receptors for this study (Figure 6). Using an OA-induced mitophagy model, we examined the effect of UBE4B on these interactions via co-immunoprecipitation. The results demonstrated that the binding affinity between NIPSNAP1 and NDP52 was significantly enhanced in both cell lines upon UBE4B overexpression. Similarly, in HEK293T cells, the interaction between NIPSNAP1 and p62 was markedly augmented under conditions of UBE4B overexpression. In contrast, in Parkin-deficient HeLa cells, although p62 expression was detectable in the input lysates, no specific binding between NIPSNAP1 and p62 was observed by co-immunoprecipitation. Previous studies using both in vitro and in vivo ubiquitination experiments have confirmed that the UBE4B (P1140A) mutation inhibits the ubiquitination function of UBE4B [59]. We therefore constructed the UBE4B (P1140A) mutant and found that its expression did not increase the interaction of NDP52 and p62 with NIPSNAP1. Collectively, these findings suggest that UBE4B promotes the association of NIPSNAP1 with key autophagy receptors—particularly NDP52—presumably through ubiquitination, thereby enhancing the recruitment efficiency of autophagosomes to damaged mitochondria.

## 3. Discussion

As a key regulatory protein in mitophagy, NIPSNAP1 translocates to the mitochondrial outer membrane following depolarization and recruits autophagy receptors—such as p62, NBR1, NDP52, and TAX1BP1—to guide autophagosome encapsulation of damaged mitochondria and facilitate their delivery to lysosomes for degradation [29]. This study reveals a novel regulatory mechanism of NIPSNAP1: Under mitochondrial depolarization, UBE4B enhances the recruitment of autophagy receptors p62 and NDP52 by mediating the ubiquitination of NIPSNAP1, and this recruitment of NDP52 can occur in a Parkin-independent manner.

It should be noted that the UBE4B-mediated degradation we investigated, promoted by Nipsnap ubiquitination, operates independently of the PINK1-Parkin pathway. This distinguishes it from several other forms of PINK1-Parkin-independent mitophagy that also occur in cellular systems, such as that induced by Oligomycin and Antimycin treatment. It must be emphasized that there exists another distinct and important category of Parkin-independent mitophagy pathways, namely those directly mediated by receptors localized on the mitochondrial membrane, such as Bcl-2/Adenovirus E1B 19-kDa Interacting Protein 3 (BNIP3), Nip3-like protein X (NIX), FUN14 domain-containing protein 1 (FUNDC1), etc. [60]. This pathway is typically activated under specific physiological or stress conditions, such as hypoxia. These types of pathways differ fundamentally in their regulatory mechanisms, physiological significance, and upstream signaling. The present study focuses on the Parkin-independent mitochondrial clearance mechanism initiated by acute mitochondrial damage and loss of membrane potential. Future research is warranted to further compare the molecular mechanisms and functional similarities and differences between these Parkin-independent pathways.

NIPSNAP1 and NIPSNAP2 are typically localized in the mitochondrial matrix and translocate to the outer membrane upon depolarization, where they participate in the recruitment of autophagy receptors. Here, we demonstrate that ubiquitination of NIPSNAP1 acts as a key molecular switch that enhances its binding to p62 and NDP52. Given that both p62 and NDP52 contain known ubiquitin-binding domains [6,8,61], UBE4B-mediated ubiquitination of NIPSNAP1 likely enhances its affinity for these receptors, underscoring the central role of ubiquitination in this process.

Previous studies demonstrate that during OA-induced mitochondrial depolarization, NIPSNAP1 translocates to the mitochondrial outer membrane (OMM) to initiate mitophagy [29]. As ubiquitination occurs exclusively on the OMM [19], our confirmation of NIPSNAP1 ubiquitination implies its translocation to this compartment. Upon activation, NIPSNAP1 is expected to induce mitophagy. Consequently, we propose that NIPSNAP1 is ultimately degraded via this mitophagy pathway.

Dysregulation of the PINK1–Parkin pathway impairs mitophagy and represents a key pathogenic mechanism in familial Parkinson’s disease [17]. Similarly, loss of NIPSNAP1 function disrupts the clearance of damaged mitochondria, leading to dopaminergic neuron death—a process closely associated with Parkinson’s pathology [29]. Notably, the UBE4B–NIPSNAP1 regulatory axis identified in this study operates independently of the canonical Parkin pathway. Notably, the UBE4B–NIPSNAP1 regulatory axis identified in this study operates independently of the canonical Parkin pathway. The relationship between the UBE4B-NIPSNAP1 axis and the canonical PINK1-Parkin pathway is not one of simple competition or substitution. Rather, it likely constitutes a hierarchically organized, spatiotemporally complementary, and functionally synergistic regulatory network. Although both pathways may converge on shared downstream autophagy receptors, they are distinguished by their distinct triggering signals and activation contexts. Even under conditions of PINK1-Parkin pathway inactivation, UBE4B effectively promote the degradation of NIPSNAP1 via the lysosome-dependent pathway by ubiquitinating NIPSNAP1, suggesting UBE4B as a critical regulatory factor for Parkinson’s disease and related disorders. Although UBE4B-mediated ubiquitination of NIPSNAP1 is independent of Parkin, the two pathways may converge downstream by sharing certain autophagy receptors, indicating a potential dynamic relationship of both competition and complementarity. When the PINK1–Parkin pathway is highly active, its robust ubiquitination signaling may dominate, rendering the role of UBE4B relatively secondary. However, under pathological conditions such as aging, genetic mutations, or specific cellular stresses that attenuate PINK1-Parkin function [62,63]. By ubiquitinating NIPSNAP1, UBE4B can independently promote the degradation of NIPSNAP1 via the lysosome-dependent pathway, which is likely involved in mitophagy, thereby sustaining mitochondrial quality control. Moreover, even when the Parkin pathway is functional, UBE4B may act cooperatively to enhance the recruitment of autophagy receptors and improve clearance efficiency.

Recent years have witnessed a substantial expansion in our understanding of the functional repertoire of NIPSNAP proteins. Beyond their involvement in autophagic signaling, NIPSNAP1 has been demonstrated to play critical roles in mitophagy, the regulation of apoptosis, the modulation of pain signaling, as well as in the pathogenesis of neurological disorders and cancer [28]. For example, in human colon cancer cells HCT116, serum deprivation upregulates NIPSNAP1 expression, thereby promoting cell growth and suppressing senescence [64]. Targeting NIPSNAP1 effectively affects c-Myc levels in cancer cells, reduces cell proliferation, induces cell cycle arrest, and induces cell senescence [64]. Our study demonstrates that UBE4B can regulate NIPSNAP1 through ubiquitination, suggesting that targeting NIPSNAP1 via UBE4B may offer a potential therapeutic strategy for tumor treatment. Furthermore, its intersection with viral immune evasion represents an emerging research paradigm, as viruses often hijack host organelle quality control pathways to sustain infection [65]. Against this backdrop, elucidating the regulatory mechanisms of NIPSNAP proteins is of particular importance. However, research in this area remains limited. Consequently, our discovery of UBE4B-mediated ubiquitination provides novel and significant insights into the regulation of NIPSNAP proteins.

In summary, we demonstrated a novel regulatory mode of NIPSNAP1 that UBE4B-mediated ubiquitination enhances its binding affinity for the autophagy receptors NDP52 under conditions of mitochondrial depolarization in HeLa cells, which do not express Parkin, thereby promoting the lysosome-dependent degradation pathway, and we speculate that mitophagy is therefore promoted. Our findings reveal a novel PARKIN-independent mitophagy pathway induced by Oligomycin/Antimycin treatment, in which UBE4B promotes the degradation by ubiquitinating NIPSNAP1, thereby expanding the current understanding of the mitochondrial quality control network.

## 4. Materials and Methods

### 4.1. Cell Culture and Reagents

All cell lines were purchased from the American Type Culture Collection. Human embryonic kidney 293T (HEK293T) cells (ATCC^®^ CRL-3216) and human cervical adenocarcinoma HeLa cells (ATCC^®^ CRM-CCL-2) were cultured in Dulbecco’s Modified Eagle Medium (DMEM; Gibco, Grand Island, NE, USA) supplemented with 10% fetal bovine serum (FBS; Gibco). Cells were maintained at 37 °C in a humidified atmosphere containing 5% CO_2_. The culture medium was refreshed every two days.

Reagents: Oligomycin (Calbiochem, Darmstadt, Germany, Cat. No. 495455), Antimycin A (Santa Cruz, Dallas, TX, USA, sc-202467), MG132 (MCE, Monmouth Junction, NJ, USA, HY-13259), CQ (MCE, HY-17589A), BafA1 (MCE, HY-100558).

### 4.2. Cell Transfection

Transfection was performed when cells reached approximately 70% confluence. HEK293T cells were transfected with Polyethylenimine Linear (PEI) MW40000 Kit (Yeasen, Shanghai, China, 40816ES03). Hela cells were transfected with Lipofectamine™ 3000 (Invitrogen, Carlsbad, CA, USA, cat. no. L3000001). After 8 h of transfection, the medium was replaced with fresh complete culture medium.

### 4.3. siRNA and Antibodies

All the siRNA were ordered from Sangon Biotech, Shanghai, China. The siRNA sequences used in the manuscript were below:

siUBE4B: 5′-CUGCAAUGCUGAACUUUAATT-3′

The following antibodies were used for Western blotting.NIPSNAP1 (Rabbit polyclonal, Santa Cruz, sc-515197, 1:1000), FLAG (Mouse monoclonal, Abcam, Cambridge, UK, ab49763, 1:1000), UBE4B (Rabbit polyclonal, Abcam, ab97697, 1:5000), LC3 (Rabbit polyclonal, CST, Danvers, MA, USA, 12741, 1:1000), UB (Rabbit polyclonal, Abcam, ab134953, 1:5000), Tubulin (Mouse monoclonal, Proteintech, Chicago, IL, USA, 66031-1-Ig, 1:5000), p62 (Rabbit polyclonal, CST, 5114, 1:1000).

### 4.4. Co-Immunoprecipitation (Co-IP)

Cells were lysed by radio immunoprecipitation assay lysis buffer I (Sangon Biotech, C500005-0100), containing Protease Inhibitor Cocktail (MCE; HY-K0010), PMSF (Beyotime, ST507-10 mL), 100X Phosphatase inhibitor complex I (Sangon Biotech; C500017-0001).Cell lysates (1 mg total protein) were pre-cleared with 10 μL anti-Flag M2 agarose gel (Merck; A2220-10 ML) and incubated in 4 °C for 4.5 h. Unbound proteins were washed with tris buffered saline (TBS) (Sangon Biotech, B040126-0005). The bound proteins were eluted by boiling SDS-PAGE loading buffer (with DTT, Sangon Biotech, C508320-0010) for 10 min and then analyzed by Western blotting.

### 4.5. Statistical Analysis

We used ImageJ (v1.54g) to analyze the gray intensity of protein bands in Western blotting results, comparing the gray intensity of the target protein band with the gray intensity of internal reference protein to obtain the relative gray intensity. Based on relative grayscale values, we created a bar graph using GraphPad 8 (prism 10.1.2). For Figure 3B, the relative gray intensity of UBE4B/NIPSNAP1/LC3 at different concentration points was divided by the relative gray intensity of the 0 concentration point to obtain the relative protein expression, then a line graph was created using GraphPad 8. Two-tailed Student’s t test was used to show the statistical significance of two groups, and the error bars represent SD of the mean (SD). Data are shown as mean ± SD. *n* = 3 independent replicates. *, *p* < 0.05; **, *p* < 0.01; ***, *p* < 0.001; and n.s., not significant.

### 4.6. Protein Structure Prediction and Molecular Docking

The three-dimensional structures of full-length UBE4B (UniProt ID: O95155) and NIPSNAP1 (UniProt ID: Q9BPW8) were predicted using the AlphaFold3. Protein–protein docking was performed using the HDOCK server, which utilizes a hybrid algorithm of template-based and ab initio docking. Both protein structures were prepared by removing water molecules and adding hydrogen atoms using the HDOCK built-in protocol. The protein–protein interaction interfaces were analyzed using PyMOL Molecular Graphics System (Version 2.5, Schrödinger, LLC, New York, NY, USA).

## Figures and Tables

**Table 1:** Molecular docking analysis of UBE4B with NIPSNAP1.

Model	1	2	3	4	5	6	7	8	9	10
Docking Score	−243.44	−242.46	−236.53	−232.58	−230.88	−230.35	−225.55	−224.39	−223.93	−221.65
Confidence Score	0.8663	0.8640	0.8495	0.8391	0.8345	0.8330	0.8192	0.8157	0.8144	0.8074
Ligand rmsd (Å)	51.16	79.30	45.14	50.70	49.95	54.82	52.06	45.85	42.37	44.15

## Data Availability

The original contributions presented in this study are included in the article. Further inquiries can be directed to the corresponding authors.

## Supplementary Materials

The following supporting information can be downloaded at: https://www.mdpi.com/article/10.3390/ijms27021119/s1.

## Abbreviations

CHIP	C-terminus of Hsc70-Interacting Protein
BNIP3	Bcl-2/Adenovirus E1B 19-kDa Interacting Protein 3
E6AP	E6-Associated Protein
FAT4	FAT Atypical Cadherin 4
FUNDC1	FUN14 domain-containing protein 1
HCT116	Human Colon Cancer 116
H-DOCK	Homology-based DOCKing
HEK293T	Human Embryonic Kidney 293 cells
LC3	Microtubule-Associated Protein 1A/1B-Light Chain 3 (MAP1LC3)
NBR1	Neighbor of BRCA1 gene 1 protein
NDP52	Nuclear Dot Protein 52 kDa
NIX	Nip3-like protein X
OA	Oligomycin and Antimycin A
OPTN	Optineurin
OUT	Orthogonal ubiquitin transfer
p53	Tumor Protein p53
p62	Sequestosome 1
PINK1	PTEN-Induced Kinase 1
PMSF	Phenylmethylsulfonyl Fluoride
RMSD	Root-Mean-Square Deviation
Rsp5	Reverses Spt- Phenotype 5
TAX1BP1	Tax1-Binding Protein 1
UBE4B	Ubiquitination Factor E4B
Ufd2	Ubiquitin Fusion Degradation 2
