# Supplementary material for: UBE4B Mediates Mitophagy via NIPSNAP1 Ubiquitination and NDP52 Recruitment
Source: Int J Mol Sci. 2026 Jan 22;27(2):1119. doi: 10.3390/ijms27021119 (PMC12842286; doi:10.3390/ijms27021119)
Supplement: Supplementary file 1 [file ijms-27-01119-s001.zip › ijms-4082566-supplementary.pdf]

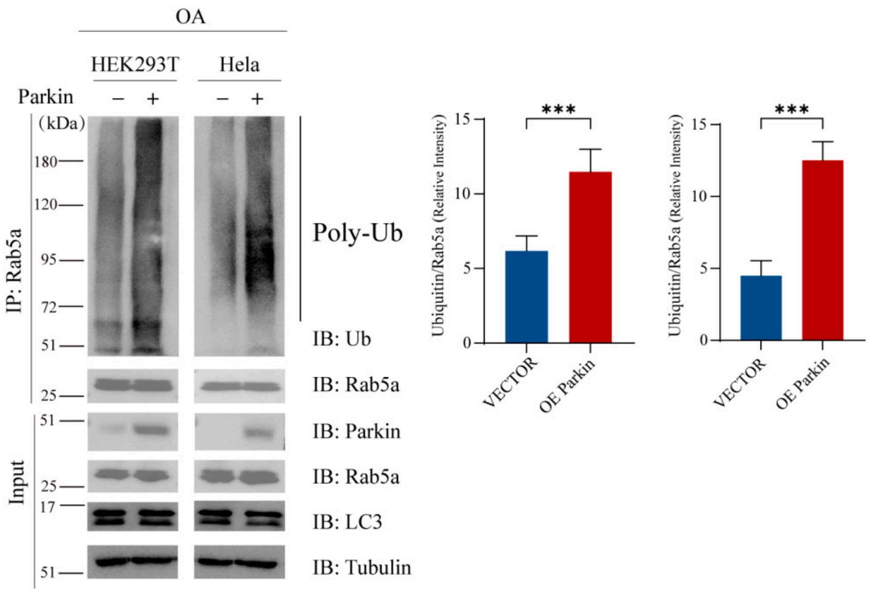

Figure S1. Parkin mediates the ubiquitination of Rab5a. HEK293T and HeLa cells are transfected with exogenous Parkin(2ug) or an empty vector(2ug) plasmid as a control for 48h and then treated with OA(10μM Oligomycin plus 4μM Antimycin-A) for 24h, together with MG132(10μM) for 4 h. Rab5a ubiquitination is detected by a coimmunoprecipitation assay with the anti-Rab5a and anti-ubiquitin antibodies. Data represent mean ± SD (n=3 independent experiments). Statistical analysis was performed using two-tailed Student's t-test, all analyses performed using GraphPad Prism 8. Statistical markers: * * * $p < 0.001$.
